# A Phosphate-Modified Aqueous Acrylic–Alkyd Resin for Protective Technology to Prevent Corrosion of Iron Substrates

**DOI:** 10.3390/polym17070847

**Published:** 2025-03-21

**Authors:** Chenglong Jiao, Wei He, Shixiong Sun, Wenhao Du, Benbo Zhao

**Affiliations:** 1 School of Chemistry and Chemical Engineering, North University of China, Taiyuan 030051, China; 2 Dezhou Industrial Technology Research Institute, North University of China, Dezhou 253034, China; 3 Shanxi North Xing’an Chemical Industry Co., Ltd., Taiyuan 030051, China; 4 State Key Laboratory of Polymer Materials Engineering, Chengdu 610065, China

**Keywords:** ferrous corrosion, phosphate group, acrylic resin, alkyd resin, water-based paint

## Abstract

Iron corrosion is very common in our daily life, and its effective protection can extend its service life. As a small molecule monomer, 2-hydroxyethyl methacrylate phosphate (HEMAP) has a phosphate group that can effectively chelate with iron ions to form a passivation layer (iron phosphate), thus slowing down the corrosion rate of iron. This study synthesized HEMAP-modified acrylic–alkyd resin copolymers with variable concentrations using free radical polymerization. The addition of HEMAP not only increases the cross-linking density of the resin, but it also further strengthens the adhesion between the resins and the iron substrate, which prevents corrosive substances from penetrating the resin. According to electrochemical studies, adding 2% mass fraction of HEMAP to the resin could greatly increase its resistance to corrosion. This study reveals HEMAP’s capacity to enhance the protection of coatings on iron substrates and lengthen the metal’s service life.

## 1. Introduction

Currently, iron-based materials have become indispensable metal materials in industrial production and human daily life [1]. H_2_O, O_2_, and Cl^−^ in environmental media can cause severe corrosion of metals, leading to huge economic losses, and are more likely to cause serious personal accidents [2,3]. Organic coatings are an effective and affordable way to protect metal surfaces because they create a barrier between the corrosive medium and the metal substrate [4,5,6,7]. For reducing the release of volatile organic compounds (VOCs), waterborne coatings like waterborne polyurethanes [8,9], waterborne epoxy resins [10], waterborne acrylates [11,12,13,14], waterborne alkyd resins [15,16], and so on, as kinds of waterborne coatings using water as a solvent, have gradually received widespread attention [17,18,19].

Alkyd resins have received wide attention for their advantages, such as good weathering resistance and low toxicity. Various acrylic monomers are copolymerized to create acrylic resins, which have strong mechanical and adhesive qualities because of their high carboxyl and hydroxyl group content, which increases their hydrogen bonding capacity [20]. Therefore, acrylic–alkyd resin, as a composite corrosion-resistant coating, effectively combines the advantages of the two, with lower cost-effectiveness, stronger mechanical strength, and better weather resistance [21,22,23]. Yan et al. [24] synthesized an aqueous acrylic resin-modified alkyd resin composite coating exhibiting excellent hardness, water resistance, and robust corrosion resistance on tinplate. Nonetheless, since the adhesion of composite resins to iron substrates is mainly a dynamic hydrogen bonding interaction, it limits their effectiveness in protecting the metal.

Phosphoric acid, as a functional monomer, can form beneficial bonds with a variety of metals, and is often used in flame-retardant and corrosion-resistant coatings [25,26,27,28]. Phosphate groups can chelate with iron atoms on the surface of the iron substrate to form a dense protective barrier and increase the strength of the bond between coating and substrate [12,29,30,31]. The tighter the bond between the coating and the metal, the more difficult it is for corrosive liquids to penetrate the coating/substrate contact, thus increasing the resistance of the coating to corrosion [32]. In a prior study, Chaudhari et al. [33] integrated phosphorylated lignin with epoxy-based coatings and assessed the corrosion protection capabilities of these coatings on tinplate substrates, revealing that the phosphoric acid moiety in conjunction with iron generated iron phosphate, which effectively impeded the penetration of corrosive agents. A parallel study by Wang et al. [12] demonstrated the efficacy of phosphate-modified coatings through their development of 2-hydroxyethyl methacrylate phosphate (HEMAP). When incorporated into traditional aqueous acrylic–epoxy systems by means of free-radical polymerization, the formation of iron phosphate complexes enhances the protective properties of the coatings. These findings align with broader research indicating that phosphoric acid group incorporation significantly bolsters the anti-corrosive performance of resin-based protective coatings.

In the present study, alkyd resins were first produced by a polycondensation reaction, and then a double bond was grafted onto the alkyd resins’ side links using acrylic acid. Phosphate-modified acrylic–alkyd resin copolymers were made by reacting various acrylic monomers and 2-hydroxyethyl methacrylate phosphate (HEMAP) with them through free radical copolymerization reaction. The corrosion resistance characteristics of various resins were examined by varying the quantity of HEMAP included. It utilizes the chelating effect of phosphoric acid molecules on iron atoms to enhance the bonding strength between the resin and the iron substrate, which can effectively resist corrosive media and extend the service life of the metal.

## 2. Materials and Methods

### 2.1. Materials

Reagents were sourced from multiple suppliers: Tianjin Damao Chemical Reagent Factory (Tianjin, China) (trimethylolpropane, TMP; phthalic anhydride, PA), Anhui Ruifende Fats and Oils Deep Processing Co., Ltd. (Xiancheng, China) (industrial-grade linoleic acid, LA), and Guangzhou Jingde Chemical Materials Co., Ltd. (Guangzhou, China) (2-Hydroxyethyl Phosphate Methacrylate, HEMAP). Shanghai McLean Biochemical Technology Company Limited (Shanghai, China) provided methyl methacrylate (MMA), acrylic acid (AA), butyl acrylate (BA), and hydroxyethyl acrylate (HEA). N-Dodecanethiol was obtained from Shanghai Eon Chemical Technology Co., Ltd. (Shanghai, China), while ethyl acetate (EAC) and benzoyl peroxide (BPO) were supplied by Fuchsin (Tianjin) Chemical Reagent Co., Ltd. (Tianjin, China) All chemicals were of analytical grade, except for LA.

### 2.2. Alkyd Resin Formulation

**Scheme 1:**
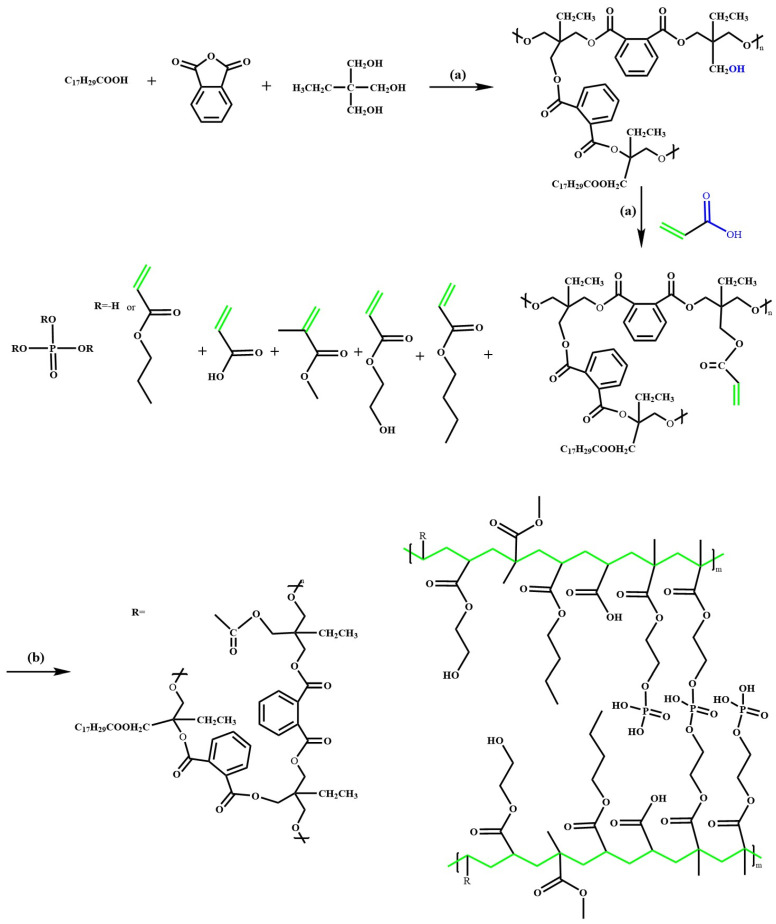
Synthesis route of HEMAP-modified acrylic–alkyd copolymer: (**a**) Preparation route of alkyd resin; (**b**) Preparation route of HEMAP-modified acrylic–alkyd resin.

The pathway for the manufacture of alkyd resins is delineated in Scheme 1a. A four-necked flask equipped with a condensing device contained xylene, TMP, LA, and PA, and the temperature was gradually elevated to 210 °C for a duration of 4 h. Thereafter, the temperature was lowered to 180 °C, and AA was introduced for 1 h. The xylene was subsequently distilled to obtain the alkyd resin.

### 2.3. Synthesis of HEMAP-Modified Acrylic–Alkyd Resin Copolymers

As depicted in Scheme 1b, the synthesis of HEMAP-modified aqueous acrylic–alkyd resin began with combining several key components: BA, HEA, MMA, alkyd resin, n-dodecanethiol, BPO, and EAC in a beaker. Following thorough homogenization, the mixture was divided, with one-fifth directly appended to a flask held at 85 °C, while three-fifths was transferred to a constant-pressure funnel for controlled addition to the reaction vessel. Subsequently, AA and HEMAP were included into the remaining two-fifths of the solution, mixed thoroughly, and then poured into the constant pressure funnel. Finally, the aqueous solution of HEMAP-modified acrylic–alkyd resin was prepared by lowering the temperature to room temperature, neutralizing with the addition of TEA, and aqueous emulsification with the addition of deionized water under high-speed stirring for 30 min. For convenience, the resulting product was named ALAPx (x indicates the mass ratio of HEMAP in the copolymer to the resin).

### 2.4. Process of Preparing Resin Film

The prepared resin was poured into a PTFE mold and evaporated at 25 °C for 3 days. The HEMAP-modified acrylic–alkyd resin adhesive film could be obtained by waiting for the gradual evaporation of the water in the resin.

### 2.5. Formulation of Resin Paint Film

Following the directives of GB/T 1727-2021 [34], the resin was uniformly applied to the tinplate substrate using a 30 μm line stick applier, and thereafter, baked in the dry box at 30 °C for 24 h, leading to a fully dried resin layer of approximately 5 μm thickness. Tinplate, the main component of which is iron, was polished with fine sandpaper before use to a roughness of Ra 0.4–1.6 µm.

### 2.6. Characterization

Surface characterization of the resins was performed using multiple analytical techniques. Spectroscopic analysis employed a Nicolet iS50 Fourier infrared spectrometer and K-Alpha X-ray photoelectron spectroscopy (both from Thermo Fisher Scientific, Waltham, MA, USA). Morphological examination was conducted via scanning electron microscopy (MERLIN Compact, Carl Zeiss AG, Oberkochen, Germany), while elemental distribution mapping utilized energy dispersive X-ray spectroscopy (MIRA LMS, TESCAN, Brno Czech, Republic). The molecular weights of different resins were measured by gel permeation chromatography (GPC) (LC-20ADXR, Shimadzu Corporation, Kyoto, Japan) with tetrahydrofuran as the mobile phase at a flushing rate of 1 mL/min. The ^31^PNMR spectra of the resins were measured using a liquid nuclear magnetic resonance spectrometer (Ascend TM 600 MHz, Bruker Corporation, Bremen, Germany) with deuterated DMSO as solvent.

### 2.7. Water Resistance Test of Resin Adhesive Film

The adhesive film’s water contact angle was measured with a contact angle meter (SL-200, Shanghai Solon Information Technology Co., Shanghai, China). The cured resin gel film was submerged in deionized water for 24 h at ambient temperature, and the absorption rate is determined by Formula (1):

(1) $$\mathrm{Water}\;\mathrm{absorption}=\frac{W-W_{0}}{W_{0}}\times 100\%$$

where “$W_{0}$” and “W” denote initial and end mass of the film, respectively. The surface of the half of the painted film immersed in deionized water for 48 h was then recorded.

### 2.8. Thermal Stability and Dynamic Thermo-Mechanical Analysis Tests of Resin Adhesive Films

The TGA 3^+^ Thermo Gravimetric Analyser (Mettler-Toledo International, Columbus, OH, USA) was used to assess the samples’ heat stability in the 30–600 °C range and test atmosphere of N_2_ with a temperature increase rate of 10 °C/min.

### 2.9. Electrochemical and Salt Spray Testing of Paint Samples

The electrochemical data of different resin paint film samples on tinplate were tested using an electrochemical workstation (CH1760E, Beijing, China) in accordance with the GB/T 29088-2012 standard [35]. The electrolyte was a sodium chloride solution of 3.5% by mass. The operating electrode was a 1 cm^2^ sample of the paint film under examination, while the secondary and parameter electrodes were platinum and calomel saturation electrodes, respectively. Polarization tests were executed at room temperature with a scan rate of 0.1 mV/s. Electrochemical Impedance Spectroscopy (EIS) measurements were performed with a scan rate of 10 mV/s for three scans. The samples were also subjected to salt spray testing using a salt spray test chamber (TYW, Wuxi Helix Environmental Equipment Co., Wuxi, China) at room temperature.

## 3. Results

### 3.1. Substance and Chemical Composition of the Resin Film

**Figure 1:**
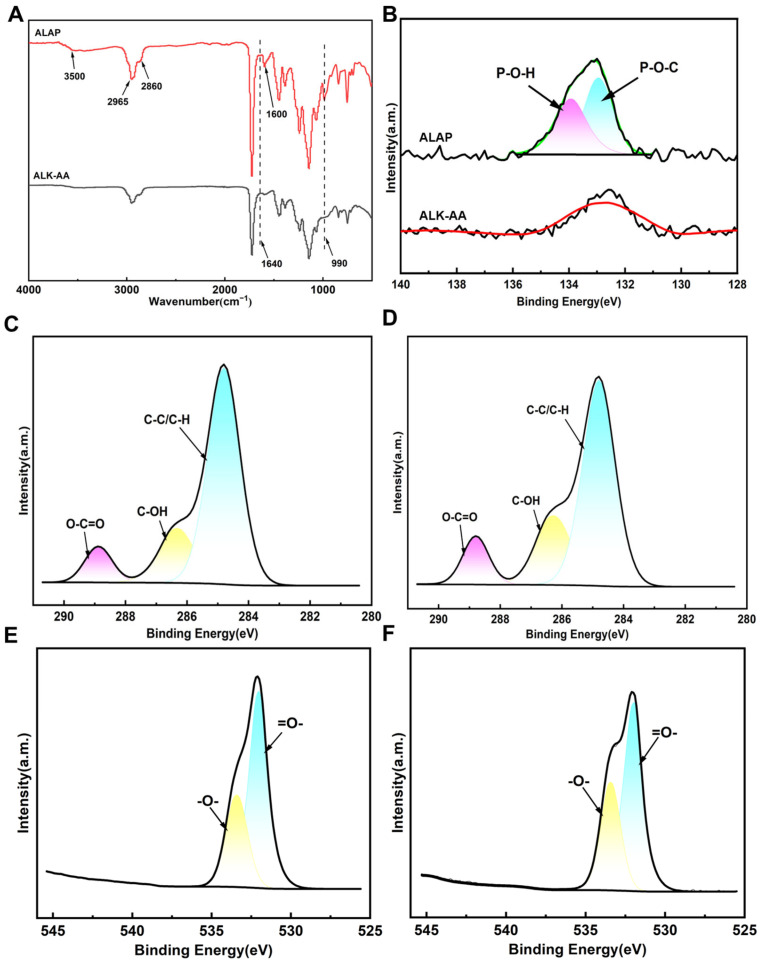
Physicochemical properties of the resins: (**A**) FTIR spectra of ALK-AA and ALAP resins; (**B**) P2p XPS spectrum of the resin; C1s spectra of ALK-AA (**C**) and ALAP (**D**); O1s spectra of ALK-AA (**E**) and ALAP (**F**).

Figure 1 presents the physicochemical analysis of ALK-AA and ALAP resins. The FTIR spectra (Figure 1A) reveal characteristic absorption bands: -OH group tensile vibrations at 3500 cm^−1^, symmetric -CH_2_ stretching at 2875 cm^−1^, asymmetric -CH_3_ tensile vibrations at 2930 cm^−1^, and aromatic C=C stretching vibration at 1600 cm^−1^ [36]. The band at 1640 cm^−1^ corresponds to olefinic C=C stretching vibrations [37]. Both synthesized resins show minimal absorption peaks, suggesting successful free radical polymerization and monomer consumption. The P-O-C extension oscillation at 990 cm^−1^ [38] appears more prominently in the HEMAP-modified acrylic–alkyd resin compared to ALK-AA, confirming successful phosphate modification.

The P2p XPS spectrum (Figure 1B) of ALAP resin displays characteristic split peaks at 133.9 eV and 132.9 eV, designated to P-O-C and P-O-H bonds from HEMAP, respectively [30]. C1s spectra analysis (Figure 1C,D) reveals similar carbon valence states in both resins, predominantly comprising C-H, C-OH, C-C, and -C=O bonds derived from acrylic–alkyd components. O1s spectra (Figure 1E,F) show two distinct bands: one at 533.4 eV corresponding to single-bonded oxygen groups (C-OH, C-O-C, and C-O-P), and another at 531.9 eV representing double-bonded oxygen species (C=O and P=O) [38]. In conclusion, the HEMAP-modified acrylic–alkyd resins have been successfully prepared, as shown by a combination of FTIR and XPS spectroscopy.

**Figure 2:**
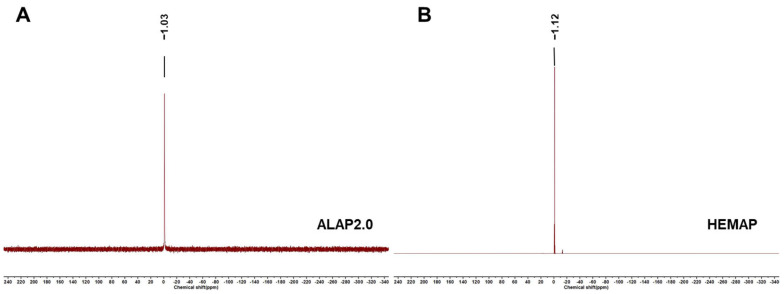
The ^31^PNMR spectra of ALAP2.0 (**A**) and HEMAP (**B**).

The 31PNMR spectra of ALAP2.0 and HEMAP are shown in Figure 2. The stronger single peak in Figure 2B (δ = −1.12 ppm) corresponds to the chemical shift of P atoms in HEMAP [39,40], whereas in ALAP2.0, there is a small shift (δ = −1.03 ppm) due to the shielding effect of the alkyd resin and acrylic resin. This indicates that HEMAP was present in the prepared ALAP2.0 samples and that the P element in HEMAP did not change its chemical state.

The weight-average molecular weight (Mw), number-average molecular weight (Mn), and polydispersity index (PI) of different resins measured by gel permeation chromatography (GPC) are shown in Table 1. With the increasing HEMAP content, the Mw and Mn of the resins showed a gradual increase, indicating that HEMAP can enhance the degree of cross-linking of the resins. As an important parameter to measure the uniformity of particle size distribution of the material, a smaller PI value indicates that the emulsion particle size distribution is more uniform. As the content of HEMAP increases, the PI of the resin is smaller, which indicates that HEMAP as a cross-linking agent reduces the self-polymerization of acrylic monomer and the emulsion distribution is more uniform.

### 3.2. Microscopic Morphology of Resins

**Figure 3:**
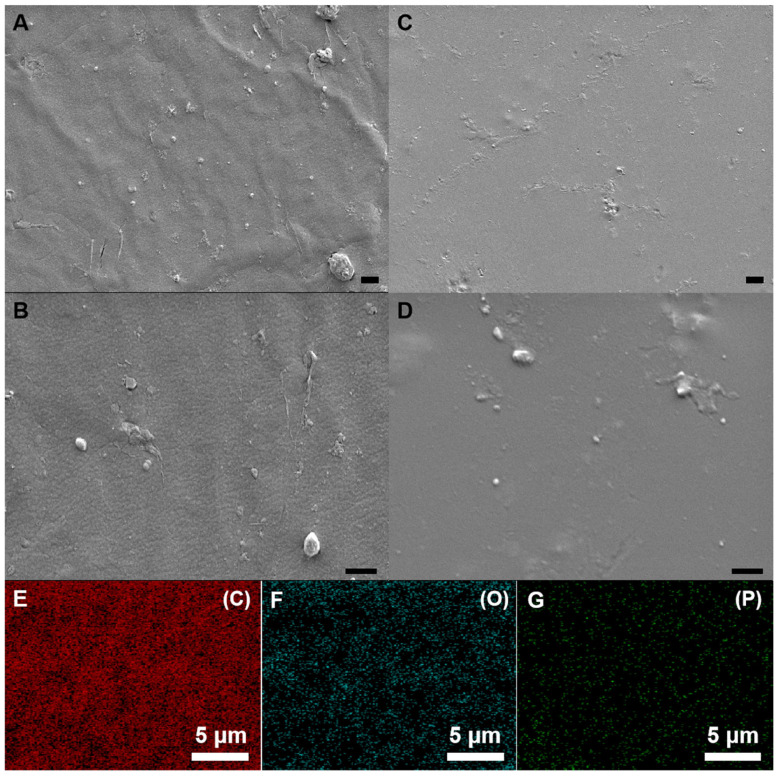
Surface morphology of the resins: SEM images of the ALK-AA resin surface magnified 5000× (**A**) and 10,000× (**B**) (scale bar 1 μm); SEM images of the ALAP resin surface magnified 5000× (**C**) and 10,000× (**D**) (scale bar 1 μm); and EDS mapping of the C (**E**), O (**F**), and P (**G**) elements of the ALAP resin face.

The SEM pictures of ALAK-AA resin are displayed in Figure 3A,B. Following 5000 and 10,000 iterations of the procedure, the resin’s surface exhibits a rougher and uneven shape. On the other hand, ALAP resin has a smoother surface with less aggregated particles (Figure 3C,D). It is possible that the tail of the HEMAP-modified resin chain will provide phosphate groups, which enhance the polarity of the resin to some extent. This makes the copolymer better dispersed in water solvent. A more ideal coating can be formed during the copolymerization process if the resin’s degree of cross-linking is enhanced by the two double bonds present in some HEMAP. The EDS mapping was conducted on the surface of the resin using an accelerating voltage of 15 kV and a resolution of 2 nm, and the distribution of C, O, and P elements on the surface of the ALAP resin is shown in Figure 3E–G. The distribution of C, O, and P elements on the surface of the ALAP resin is shown in Figure 3E–G. Among them, the C element has the highest distribution, followed by O element, and the P element has the lowest distribution; and the main chains of acrylic resins in the resin are mainly connected in the form of carbon chains, while a smaller amount of C-O bonds on alkyd resins. HEMAP, with the P element, has the lowest amount of addition. All three elements are evenly distributed on the surface of the coating, which indicates that HEMAP is evenly distributed in the ALAP resin.

### 3.3. Thin Film Properties of Samples

Table 2 summarizes the basic properties of different resin coatings. For a coating to have good corrosion resistance, it must have strong adhesion properties to ensure that the resin adheres firmly to the substrate and prevents penetration of corrosive media. All of the resins in Table 2 show good adhesion to the substrate, reaching level 0. At the same time, the hardness of the coatings increased with the increase in HEMAP: this is because HEMAP can increase the cross-linking density within the resin, which improves the hardness of the coatings to a certain extent. This means that the resin modified by HEMAP will have strong durability and improve the service life of the coating. Meanwhile, the drying time and the real drying time of all the samples were within 30 min and 2 h, respectively, which were similar to the drying times of the coatings in other studies. This indicates that the addition of HEMAP does not increase the drying time of the resin coatings, which will avoid the cost of the coatings due to the long drying time in practical applications.

### 3.4. Water-Resistance Analysis

**Figure 4:**
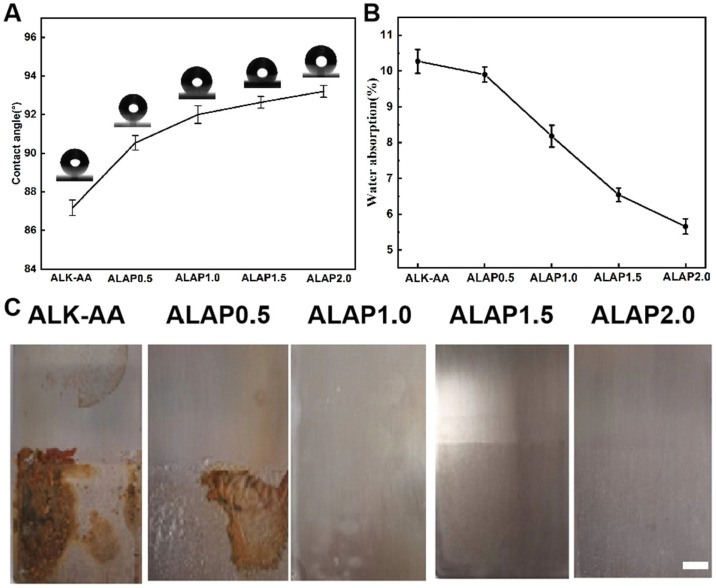
Water resistance of resin samples: (**A**) water contact angle of different resin adhesive films; (**B**) absorbency of the samples; (**C**) pictures of the samples immersed in water for 24 h (scale bar 1 cm).

Figure 4 illustrates the water resistance of various resin samples. Figure 4A illustrates that the water contact angle on the resin surface progressively rises with the augmentation of HEMAP content. The water absorption of the resin diminishes as HEMAP content increases, as illustrated in Figure 4B, indicating that HEMAP enhances the resin’s water resistance. HEMAP increases the cross-link density of the resin, attributing to a denser network structure that efficiently inhibits water molecules from penetrating the resin. The waterproof image of the resin sheet in Figure 4B further substantiates this more intuitively. As HEMAP content increases, the blistering and corrosion phenomena on the film’s surface progressively diminish. This is partly due to the increase in cross-linking degree, and partly due to the chelation of phosphoric acid with iron ions, which makes the coating more firmly bonded to the iron substrate.

### 3.5. Thermal Stability of Resins

**Figure 5:**
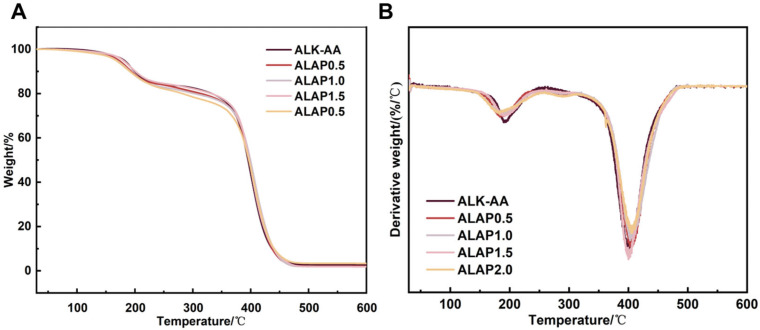
TGA curves (**A**) and DTA curves (**B**) for different resins.

The thermal stability of the resin films was monitored by thermogravimetric analysis (TGA) and the mass change curves of the samples with temperature (TG) and differential thermogravimetric profiles (DTG) were obtained as shown in Figure 5. The thermal weight loss curves of all the resins were approximately similar and basically divided into two phases, which can be observed in Figure 5B. The initial phase at 0–100 °C represents the evaporation of water from the polymer. As the temperature increases, the water in the polymer gradually evaporates into the air, which occupies a smaller mass of the resin. The stage around 400 °C is associated with thermal degradation of the polymer [41] and this loss of mass accounts for the major part of the mass of the resin. At this stage, the chemical bonds of the polymers start to break gradually, forming small molecular structures that volatilize into the air, indicating that the resins have a certain degree of human stabilization at 400 °C. The TGA and DTA curves confirm that all the resins start to decompose at about 400 °C, which provides a wide temperature range for coating applications.

### 3.6. Analysis of Corrosion Resistance of Resins

**Figure 6:**
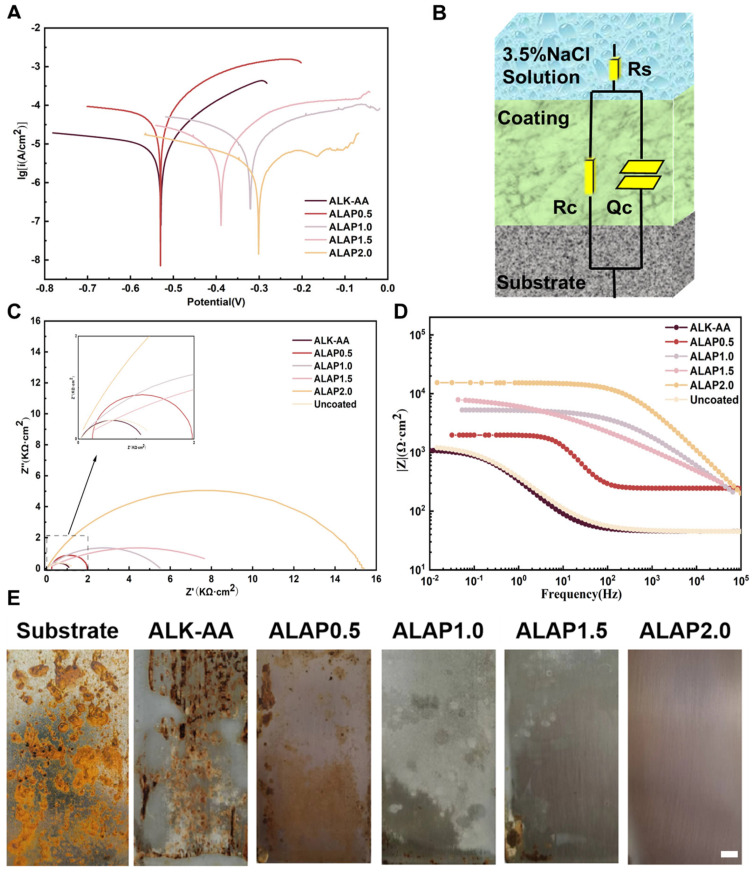
Corrosion resistance of the resins: (**A**) Polarization curves of the resins; (**B**) Electrochemical equivalent circuit diagrams used for fitting; Nyquist plots (**C**) versus Bode plots (**D**) of the different resins; (**E**) Images of the different resins after 7 days of neutral salt spray testing (scale bar 1 cm).

The corrosion resistance of the resins is shown in Figure 6. The polarization curves of the different resins are shown in Figure 6A. Numerous studies indicate that a larger corrosion potential (E) with a smaller corrosion current density (I) represents a better corrosion resistance of the coating [3,42,43]. Among all the coatings, the ALAP2.0 sample has the largest E and the smallest I. This means that the coating has good corrosion resistance in corrosive media. This means that the coating is less prone to lose electrons in corrosive media and has a smaller corrosion rate, which provides greater corrosion resistance. This should be ascribed to the chelation of the phosphate groups with the iron atoms of the substrate, resulting in the formation of iron phosphate between the iron substrate and the coating [33]. Therefore, as the HEMAP content increases, the bond between the coating and the substrate becomes denser and more resistant to corrosive media.

EIS is an important tool for qualitative and quantitative testing of the corrosion resistance of coatings, and it provides a sensitive indication of the structural changes at the coating–metal interface and the damage process at the coating interface [2,5,42,44,45]. The EIS data for the different resins were fitted using the equivalent circuit diagrams of Figure 6B to produce Nyquist plots (Figure 6C) and Bode plots (Figure 6D). In the Nyquist plots, all resins exhibit a semicircle (the capacitive loop), which corresponds to the coating resistance (Rc) in parallel with the coating capacitance (Qc). A larger diameter indicates a better corrosion resistance of the coating. It can be seen from Figure 6C that the radius of the capacitive arc becomes larger and larger as the content of HEMAP increases. It shows that HEMAP can effectively enhance the protection of the resin on the substrate and prevent the contact of corrosive media with the substrate, and the component with the highest content, ALAP2.0, has the strongest corrosion resistance. In the Bode plot, the high-frequency region indicates the capacitive properties and microporous resistance, and the low-frequency part reflects the corrosion response of the metal coating interface. The low frequency (|Z|_0.01Hz_) is often considered a semi-quantitative indicator of a coating’s corrosion resistance, with larger |Z|_0.01Hz_ indicating better corrosion resistance [7]. As the HEMAP content increases, the corrosion resistance of the coating becomes better and better. Meanwhile, the fitted impedance parameters, solution resistance (Rs), coating capacitance (Qc), coating resistance (Rc), and the chi-squared value (X^2^), were obtained as shown in Table 3. In the ZsimpWin 3.6 software, X^2^ < 5 × 10^−3^ indicates that the fitted data are reasonable [46]. The X^2^ of all coatings fitted was below 5 × 10^−3^, indicating that the electrochemical parameters fitted using this circuit diagram were accurate. The results show that ALAP2.0 has the smallest Qc (2.505 × 10^−7^ farad) and the largest Rc (1.537 × 10^4^ ohm), indicating that the corrosive medium diffuses from the solution to the metal substrate in a longer path and the resin has the most optimal corrosion protection. The pictures of the resins in Figure 6E after 7 days in a neutral salt spray environment are consistent with the EIS data of the different resins. Compared to the uncoated iron substrate material, the surfaces treated with the different resins showed less corrosion rust stains and all of them demonstrated a certain degree of corrosion resistance. The ALAP2.0 resin has the least amount of surface rust, which indicates that it has the best protection for the substrate and the resin has the best corrosion resistance.

### 3.7. Resin Anti-Corrosion Mechanism Investigation

**Figure 7:**
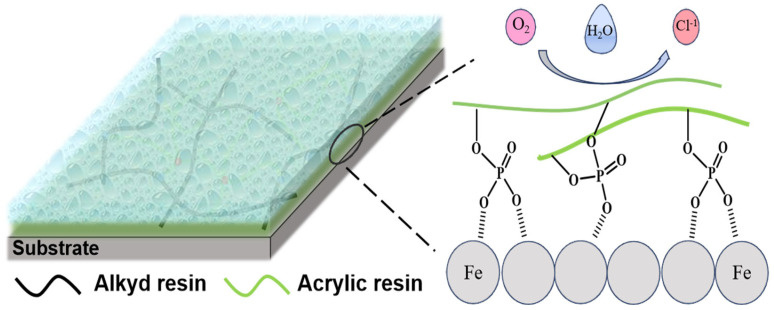
Corrosion prevention mechanism of HEMAP-modified acrylic–alkyd resin.

The hypothetical anti-corrosion mechanism of the coating is shown in Figure 7. In the natural environment, H_2_O and O_2_ molecules, as well as Cl^−^ ions, gradually penetrate between the coating and the metal by osmosis, resulting in redox reactions of iron atoms with water and oxygen, which leads to corrosion of the metal at a macroscopic level. To prevent the erosion of corrosive media, the connection between the coating and the metal substrate should be dense enough to reduce the contact between the substrate and the corrosive media. In this study, we designed and used HEMAP to modify the acrylic–alkyd resin to produce a coating with certain corrosion resistance. Polarization curves, EIS data, and salt spray resistance pictures show that the corrosion resistance of the resin is effectively improved with the increase in HEMAP content. The schematic diagram in Figure 6 explains the principle of HEMAP enhancing the corrosion resistance of the resin from a molecular point of view. Numerous studies have shown that the phosphate group of HEMAP can chelate with iron atoms, forming a layer of iron phosphate film on the iron substrate to block the intrusion of corrosive media, which improves the corrosion resistance of the coating on the iron substrate [12,30,31,47]. Therefore, as the content of HEMAP increases, the number of phosphate groups in the resin will gradually increase, and the number of chemical bonds formed between the phosphate groups and the iron atoms on the surface of the iron substrate will also increase, resulting in a stronger bond between the resin and the substrate. On the other hand, since some HEMAPs contain two double bonds, an increase in their content will also increase the cross-linking degree of the resin to a certain extent, allowing the resin to form a denser film during the curing process. Combining these two aspects, the introduction of HEMAP not only improves the bond strength between the resin and the substrate, but also makes the surface of the coating denser and reduces the erosion of corrosive media on the substrate, thus improving the corrosion resistance of acrylic–alkyd resins and prolonging the service life of metals. Due to the chelating effect of HEMAP on iron atoms, it is able to form a dense iron phosphate film on the surface of iron and its alloys, which provides effective protection for iron and its alloys.

## 4. Conclusions

In the present work, HEMAP was introduced into acrylic–alkyd resins through free group polymerization reactions to produce coatings with a certain level of corrosion resistance. The addition of HEMAP increased the degree of cross-linking in the resin, which led to the formation of a copolymer with a more compact network. As a consequence, the water resistance of the resin samples was improved to some degree. Because of the presence of the phosphoric acid group in HEMAP, the chelating ability between the resin and the tinplate substrate is increased, which in turn strengthens adhesion at the interface between the coating and the iron foundation plate. This makes it more difficult for the electrolyte solution to access the coating/iron contact, which ultimately results in an increase in the coating samples’ resistance to corrosion. With the addition of a 2% mass fraction of HEMAP, the corrosion resistance of the modified acrylic–alkyd resin is shown to reach its maximum level, as demonstrated by the findings of the EIS. There was an improvement in the qualities that prevent rusting. This study demonstrates that HEMAP-modified acrylic–alkyd resins considerably improve the protective properties of coatings. As a result, this phosphoric acid-modified alkyd resin may be a means of addressing corrosion of ferrous substrates.

## Figures and Tables

**Table 1:** Mn, Mw, and PI values for different resins.

Sample	Mw (g/mol)	Mn (g/mol)	PI
ALK-AA	96,427	42,302	2.279
ALAP0.5	116,147	55,028	2.111
ALAP1.0	126,170	63,986	1.972
ALAP1.5	139,856	75,975	1.841
ALAP2.5	157,601	96,274	1.637

**Table 2:** Fundamental characteristics of the coatings on different samples.

Sample	Adhesion	Pencil Hardness	Dry-to-Touch Time (min)	Dry-Hard Time (h)
ALK-AA	0	HB	<30	<2
ALAP0.5	0	HB	<30	<2
ALAP1.0	0	H	<30	<2
ALAP1.5	0	H	<30	<2

**Table 3:** Nyquist data for different resins.

Sample	R_S_, ohm	Qc, farad	Rc, ohm	X^2^
Uncoated	45.14	7.054 × 10^−4^	1268	4.07 × 10^−3^
ALK-AA	45.33	7.128 × 10^−4^	1082	3.86 × 10^−3^
ALAP0.5	245.8	1.117 × 10^−5^	1726	4.77 × 10^−3^
ALAP1.0	1.037 × 10^−4^	2.02 × 10^−6^	5527	1.08 × 10^−3^
ALAP1.5	1.299 × 10^−3^	2.184 × 10^−5^	8826	2.47 × 10^−3^
ALAP2.0	1.891 × 10^−6^	2.505 × 10^−7^	1.537 × 10^4^	1.93 × 10^−3^

## Data Availability

The original contributions presented in this study are included in the article. Further inquiries can be directed to the corresponding author.
